# A Comparative Benchmark of Modern Deep Learning Backbones for Multi-Class Periodontal Bone Loss Staging

**DOI:** 10.3390/jcm15218644

**Published:** 2026-11-06

**Authors:** Nurullah Duger, Burak Dagtekin, Furkan Talo, Melek Atille Aydin, Mucahit Karaduman, Muhammed Yildirim, Tuba Talo Yildirim, Tolga Fikret Tozum

**Affiliations:** 1 Department of Periodontology, Faculty of Dentistry, Firat University, Elazig 23100, Turkey; bdagtekin@firat.edu.tr (B.D.); matille@firat.edu.tr (M.A.A.); taloyildirim@firat.edu.tr (T.T.Y.); 2 Digital Transformation and Software Office, Firat University, Elazig 23100, Turkey; ftalo@firat.edu.tr; 3 Department of Software Engineering, Malatya Turgut Ozal University, Malatya 44200, Turkey; mucahit.karaduman@ozal.edu.tr; 4 Department of Artificial Intelligence and Data Engineering, Firat University, Elazig 23119, Turkey; muhammedyildirim@firat.edu.tr; 5 Department of Periodontics, College of Dentistry, University of Illinois Chicago, Chicago, IL 60607, USA; ttozum@icloud.com

**Keywords:** periodontitis, alveolar bone loss, machine learning, artificial intelligence, computer-assisted diagnosis

## Abstract

**Background/Objectives:** Early diagnosis of periodontal disease is critical to prevent tissue destruction and tooth loss. This study evaluates six modern deep learning architectures for four-class radiographic periodontal bone loss grading on panoramic radiographs. **Methods:** This retrospective study analyzed 1053 images. Two periodontologists independently classified each image according to the most severely affected tooth into four bone loss categories: Healthy, Mild (<25%), Moderate (25–50%), and Severe (>50%). Six convolutional, transformer, and hybrid backbones (ConvNeXt-Tiny, MaxViT-Tiny, EfficientViT-B1, FocalNet-Tiny, CAFormer-S18, and DaViT-Tiny) were trained on a 70/10/20 stratified split using an NVIDIA Tesla T4 GPU, the AdamW optimizer, Cosine Annealing, and early stopping. Performance was assessed using standard classification metrics, Matthews Correlation Coefficient, Cohen’s and Quadratic Weighted Kappa, Expected Calibration Error, Area Under the Curve (AUC), McNemar’s test, and bootstrap 95% confidence intervals. **Results:** FocalNet-Tiny achieved the highest numerical scores among the tested models, reaching 83.41% accuracy, 84.70% balanced accuracy, 83.50% macro-F1, and 0.9610 OvR AUC. The *p*-values obtained from the comparisons of the models were all above 0.05, indicating that the classification performances of the evaluated models are quite similar. Bootstrap analysis confirmed stable prediction metrics for the top-scoring model. **Conclusions:** This study provides a controlled benchmark of modern deep learning backbones for four-class radiographic periodontal bone loss grading. FocalNet-Tiny achieved the highest numerical metrics and stable bootstrap estimates, yet performed comparably to other contemporary architectures such as ConvNeXt-Tiny. Direct radiographic classification shows promise for automated radiographic pre-screening; however, multi-center external validation and integration with clinical periodontal parameters are required before clinical implementation.

## 1. Introduction

Periodontal diseases, which are among the most common chronic inflammatory conditions worldwide, significantly affect both oral and general systemic health [1]. Periodontitis, consistently associated with systemic conditions such as cardiovascular diseases, type 2 diabetes, and adverse pregnancy outcomes, is characterized by the progressive destruction of the gingiva, periodontal ligament, and alveolar bone [2,3]. Given the progressive nature of the disease, early and accurate diagnosis is essential to halt tissue destruction and prevent tooth loss. Delayed intervention substantially reduces treatment success and necessitates more complex, costly procedures [4,5].

The diagnosis of periodontitis is based on the measurement of clinical attachment loss and probing depth parameters with a periodontal probe, alongside the evaluation of radiographic images demonstrating alveolar bone loss [6]. In this diagnostic process, radiological data hold a crucial place in determining the severity and extent of tissue destruction necessary for staging the disease and planning treatment. Panoramic radiographs allow for comprehensive monitoring of changes in alveolar bone levels by visualizing the entire dentition in a single image [7]. However, subjective interpretation and low inter-observer reliability in manual radiographic evaluation can limit diagnostic consistency [8]. Deep learning algorithms help overcome these limitations by analyzing complex radiographic patterns with high objectivity and reproducibility.

In recent years, deep learning methods have transformed the field of medical image analysis, and convolutional neural networks (CNNs) have become a significant area of study for automated disease detection and classification [9,10,11]. Pioneering studies conducted in the field of periodontology have proven the applicability of deep learning models to radiographic imaging. Krois et al. applied deep feed-forward CNNs to panoramic radiographs and reported a mean classification accuracy of 81% in detecting periodontal bone loss, a level comparable to the performance of six experienced dentists [8]. Chang et al. proposed a hybrid framework combining deep learning architecture with traditional computer-aided diagnosis methods to detect and stage periodontal bone loss, achieving high consistency with radiologists [12]. More recent studies have expanded the methodological repertoire; segmentation-based approaches aimed at detecting bone loss patterns and furcation defects with U-Net architectures achieved an area under the curve (AUC) value of 0.951 for total alveolar bone loss, while exhibiting lower performance for vertical defects [7]. Convolutional neural networks have also been used to classify bone loss severity levels; a representative study reported 73.0% diagnostic accuracy for the binary “normal-and-disease” task, while this rate remained at 59% for severity level classification [13]. Systematic reviews emphasize that while deep learning models yield promising results in periodontal image analysis, accuracy values vary significantly between 73.0% and 98.6%, reporting standards are inconsistent, and most studies lack statistical rigor and methodological transparency [14,15,16]. A comprehensive review examining 47 deep learning studies in periodontology and oral implantology revealed significant heterogeneity in study design and a high risk of bias in the current literature [17].

Despite the rapid diversification of deep learning architectures and the growing periodontal imaging literature, systematic comparisons of modern backbones for multi-class periodontal bone loss grading remain scarce. Furthermore, model calibration and formal statistical significance testing are rarely reported in this field. The present study addresses these gaps by evaluating the comparative efficacy of six contemporary deep learning backbones—encompassing convolutional, transformer, and hybrid families—for four-class radiographic periodontal bone loss grading on panoramic radiographs. Specifically, ConvNeXt-Tiny, MaxViT-Tiny, EfficientViT-B1, FocalNet-Tiny, CAFormer-S18, and DaViT-Tiny models, which represent different architectures and possess comparable parameters, were trained, validated, and tested under identical hyperparameter settings and data partitioning.

## 2. Materials and Methods

### 2.1. Study Design and Data Collection

This retrospective study was approved by the Fırat University Non-Interventional Clinical Research Ethics Committee (No. 2025-17-40) and was conducted in accordance with the Declaration of Helsinki. A total of 2500 panoramic radiographs obtained from patients presenting to the Fırat University Faculty of Dentistry Periodontology Clinic between January 2022 and January 2025 were reviewed. Imaging standardization was maintained by including only radiographs acquired with a Carestream Dental CS 8100 unit (68 kV, 8 mA, 66 mGy·cm^2^, 10.8 s). Localized maxillary and mandibular regions of interest (ROIs) encompassing the target teeth and surrounding alveolar bone were manually cropped from the panoramic radiographs using ImageJ software (version 1.54p, National Institutes of Health, Bethesda, MD, USA) and saved in lossless PNG format [8,9,13]. The “target teeth” were defined as the teeth within each ROI that served as the reference for grading radiographic periodontal bone loss. A tooth was considered eligible when its cementoenamel junction (CEJ), alveolar crest, and root apex could be clearly identified, so that bone loss could be assessed relative to root length. Each ROI was taken from the anterior or posterior region of one arch, whichever region was diagnostically suitable on the radiograph, and was delineated to include the crowns and entire roots of the target teeth together with the surrounding alveolar bone. This cropping step removed non-diagnostic background structures—such as the cervical spine, airway shadow, and mandibular ramus—and preserved periodontal anatomical detail at the network input resolution. Apart from manual ROI cropping, no contrast enhancement, histogram equalization, or filtering was applied prior to model training. Dataset partitioning into training, validation, and test subsets was subsequently performed on a patient basis rather than an image basis, so that all ROIs derived from the same patient, including both maxillary and mandibular crops of a single radiograph, were assigned exclusively to one subset. When multiple radiographs were available for the same patient, all of them were allocated to the same subset. This patient-level allocation ensured that no anatomical information from a given individual was shared across subsets, thereby preventing data leakage and avoiding an overestimation of model performance on the test set.

The inclusion criteria encompassed cropped panoramic ROI images from periodontally healthy individuals and patients exhibiting Mild (<25%), Moderate (25–50%), or Severe (>50%) alveolar bone loss. Patient age and sex were not restricted, ensuring a heterogeneous dataset representative of routine clinical practice. Radiographs with poor diagnostic quality due to exposure errors, motion artifacts, severe anatomical overlapping, or image distortion were excluded.

**Figure 1:**
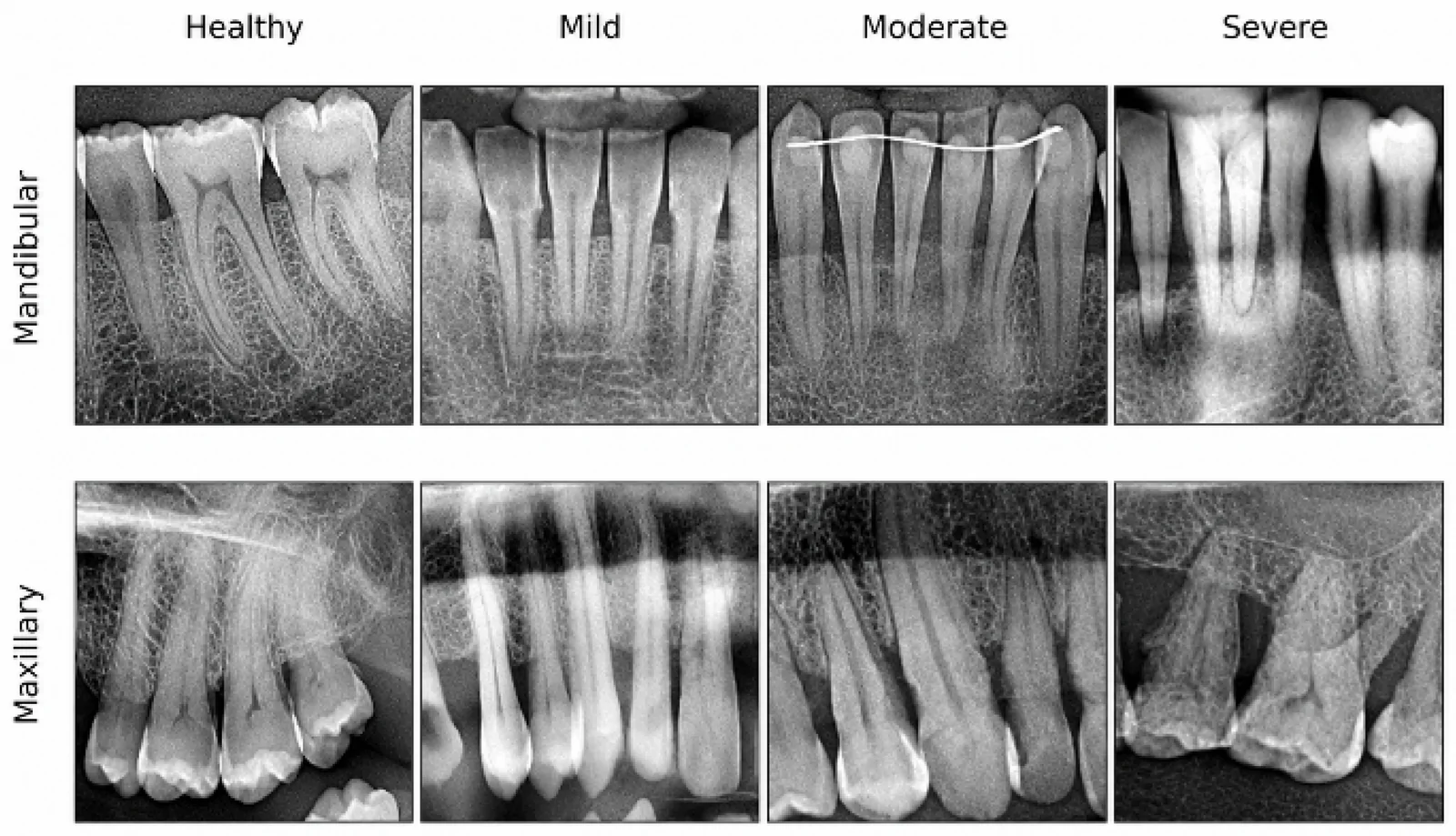
Representative cropped panoramic regions of interest (ROIs) from the mandibular and maxillary arches for each radiographic bone loss class.

A final dataset of 1053 images manually classified by two specialist periodontologists (B.D. and N.D.) was established. Representative examples for each severity class are shown in Figure 1.

### 2.2. Manual Classification and Standardization

All 1053 images were independently graded by two specialist periodontologists (B.D. and N.D.), both of whom are co-authors of the present study. Radiographic bone loss (RBL) was measured from the cementoenamel junction (CEJ) to the alveolar crest relative to the total root length, accounting for a physiological crestal distance of 1–2 mm apical to the CEJ. In images showing varying degrees of bone destruction across multiple teeth, the overall class was assigned according to the most severely affected tooth. The 2017 World Workshop classification [18] defines clinical periodontitis staging (Stages I–IV) by combining RBL with chairside parameters such as clinical attachment loss and probing pocket depth. Because concurrent clinical records were unavailable in this retrospective radiographic dataset, a four-tier radiographic grading system was adopted in line with previous deep learning benchmarks [12,13]. These four categories therefore describe the radiographic extent of alveolar bone loss and are not equivalent to the clinical stages of periodontitis. Images were categorized based on RBL relative to root length into four groups: Healthy (*n* = 218; no bone loss), Mild (*n* = 365; <25%), Moderate (*n* = 199; 25–50%), and Severe (*n* = 271; >50%). Weighted Cohen’s Kappa analysis indicated high inter-observer agreement (κ = 0.8692). Intra-observer reliability was assessed by reclassifying randomly selected images after a two-week interval, yielding κ = 0.9164. These values confirm a consistent and reproducible reference standard for model training. Disagreements between the two graders were resolved by joint re-evaluation of the image and discussion until consensus was reached, and the consensus label was used as the reference standard; no third examiner was involved.

### 2.3. Proposed Model

**Figure 2:**
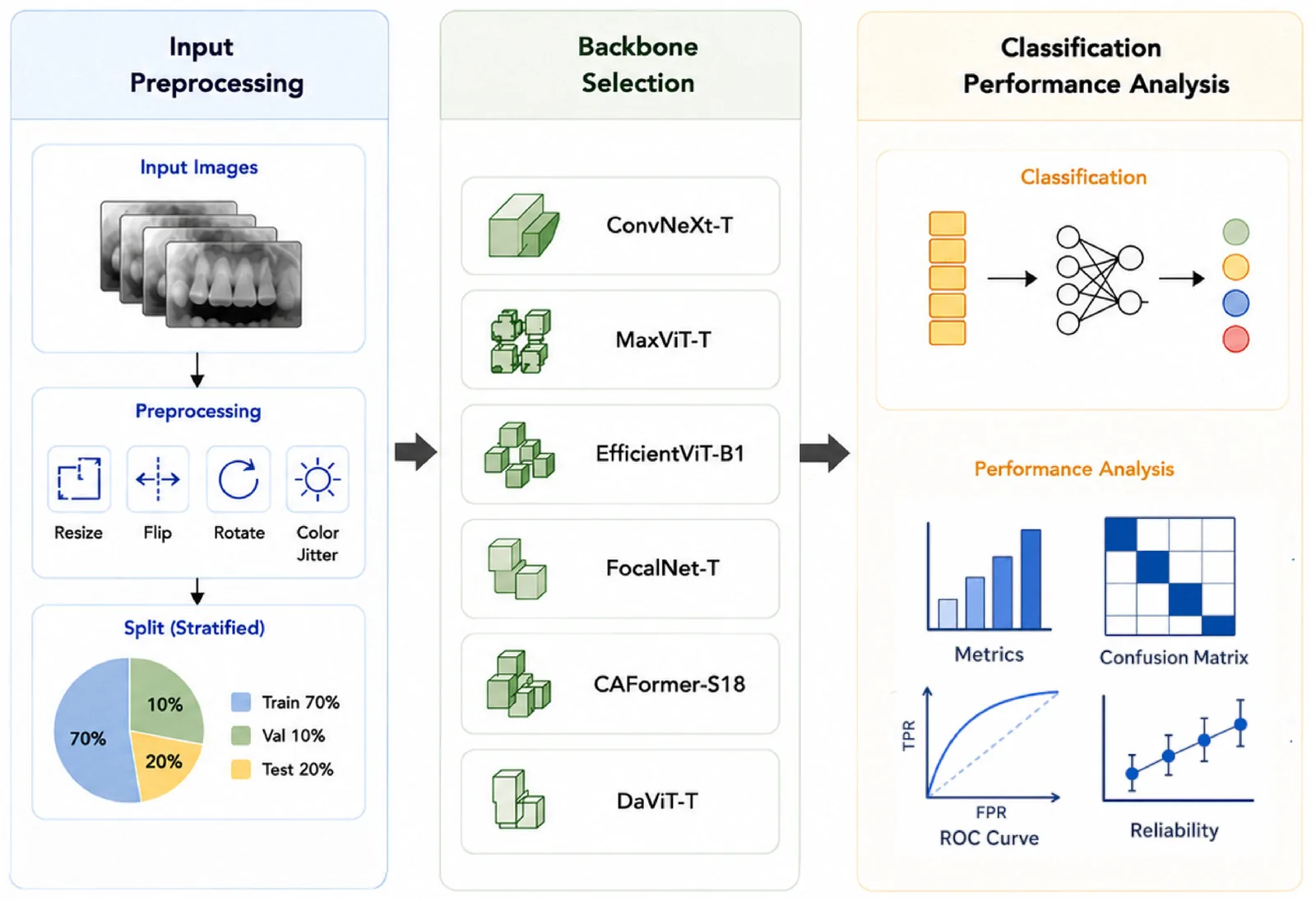
Flow diagram of the proposed method.

In this study, a backbone comparison framework based on modern deep learning architectures was developed for the automated classification of four different levels of periodontal bone loss. The primary objective of the proposed approach is to compare the performances of various contemporary image classification architectures under identical experimental conditions and determine the most suitable model for periodontal bone loss classification. The overall model workflow is provided in Figure 2. The proposed model consists of four main stages: image preprocessing, backbone architecture comparison, classification head, and performance analysis.

Six contemporary deep learning architectures representing convolutional, transformer, and hybrid families were evaluated: ConvNeXt-Tiny, MaxViT-Tiny, EfficientViT-B1, FocalNet-Tiny, CAFormer-S18, and DaViT-Tiny. ConvNeXt-Tiny is a modernized convolutional network built on contemporary design principles. MaxViT-Tiny is a hybrid transformer that combines blocked local attention and grid global attention. EfficientViT-B1 is a lightweight transformer architecture designed for high computational efficiency. FocalNet-Tiny replaces conventional self-attention with multi-scale focal modulation to aggregate local and global context. CAFormer-S18 integrates convolutional token mixers in early stages with transformer self-attention in deeper stages. Finally, DaViT-Tiny captures spatial and channel dependencies jointly through dual attention mechanisms.

All models were evaluated under the same training, validation, and testing protocol. Pre-trained weights were used in each model, and the final classification layer was adapted to the four classes in the periodontal bone loss dataset. Following feature extraction, probability values for the Healthy, Mild, Moderate, and Severe classes were generated via global average pooling and a fully connected classifier layer. The final model selection was performed based on the performance values obtained on the independent test set.

### 2.4. Experimental Setup and Training Protocol

All six architectures were trained and evaluated under identical experimental conditions. Patient-level splitting was performed to partition the 1053 ROI images into training (*n* = 737), validation (*n* = 105), and test (*n* = 211) subsets, corresponding to approximately 70%, 10%, and 20%, respectively. All images were resized to 224 × 224 pixels prior to network input. On-the-fly data augmentation—including random horizontal flipping, random rotation, and color jittering—was applied exclusively to the training subset.

Experiments were executed on an NVIDIA Tesla T4 GPU (Santa Clara, CA, USA) with a mini-batch size of 32 and the AdamW optimizer (initial learning rate = 1 × 10^−4^, weight decay = 1 × 10^−4^). A Cosine Annealing scheduler dynamically adjusted the learning rate during training. Overfitting was controlled using a multi-class Cross-Entropy loss function with a label smoothing factor of 0.05, and no class-specific weights were applied in the loss function. Early stopping was implemented with a patience of 6 epochs based on validation loss.

Each backbone was initialized with ImageNet pre-trained weights and fine-tuned for up to 15 epochs. Transfer learning from pre-trained representations enabled all evaluated architectures to converge within this 15-epoch window. Specifically, FocalNet-Tiny reached its optimal validation performance at epoch 8 and triggered early stopping at epoch 14, whereas the remaining five models ran for all 15 epochs and reached their peak validation checkpoints between epochs 10 and 15. Total parameter counts and training times were recorded to compare computational efficiency.

### 2.5. Performance Evaluation Metrics

Model performance was evaluated using overall Accuracy, Balanced Accuracy, Macro Precision, Macro Recall, Macro F1-score, and Weighted F1-score. Prediction consistency was quantified with the Matthews Correlation Coefficient (MCC), Cohen’s Kappa, and Quadratic Weighted Kappa (QWK). Multi-class discrimination was analyzed using One-vs-Rest (OvR) and One-vs-One (OvO) AUC. Model calibration was assessed via Expected Calibration Error (ECE) and multi-class Log-Loss. Confusion matrices, Receiver Operating Characteristic (ROC) curves, and bootstrap-based 95% confidence intervals (CIs) were also generated. Pairwise McNemar’s tests [19] were performed using the top-scoring model (FocalNet-Tiny) as the reference classifier to determine whether its numerical lead over the other five architectures was statistically significant.

### 2.6. Bootstrap Confidence Intervals

To quantify the uncertainty of the test-set estimates, a non-parametric bootstrap procedure [20] was applied to the top-scoring model (FocalNet-Tiny). The trained model was kept fixed throughout this analysis (no retraining), and its predicted labels and softmax probabilities for the 211 test images were stored. In each of 1000 iterations, a bootstrap sample of equal size (*n* = 211) was drawn with replacement from the test set and accuracy, macro F1-score, balanced accuracy, MCC, and OvR AUC were recomputed on each resampled set. The reported value for each metric is the mean of the 1000 bootstrap estimates, and the 95% CI was obtained using the percentile method, i.e., the 2.5th and 97.5th percentiles of the bootstrap distribution. A fixed random seed was used for reproducibility, and all computations were performed in Python (v3.15.2, Python Software Foundation, Delaware, USA). Because only the test set was resampled, these intervals reflect the sampling variability of the test data and do not capture variability arising from model training, such as random weight initialization or alternative data partitions.

## 3. Results

This section presents the comparative validation and test performances of the six evaluated architectures, followed by statistical significance testing, bootstrap stability analysis, and class-wise error evaluation.

**Figure 3:**
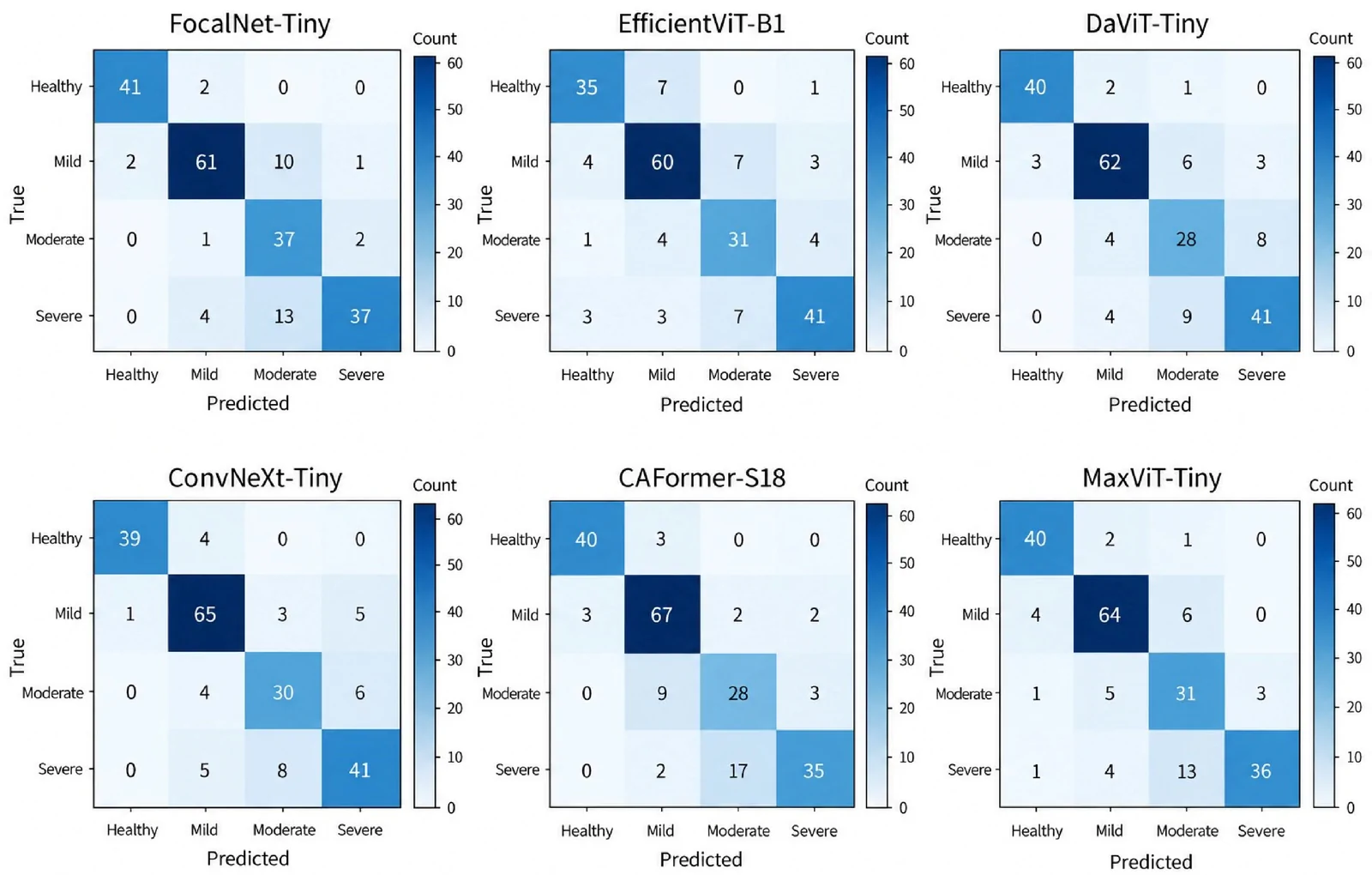
Confusion matrices of the six deep learning architectures on the independent test set (*n* = 211). Cell values indicate classified sample counts, and the vertical color bar (‘Count’, 0–60) represents sample density.

Figure 3 displays the test set confusion matrices (*n* = 211) for all six models, where each cell shows the exact sample count and the color scale (‘Count’, 0–60) reflects classification density. All architectures achieved high accuracy in the Healthy (*n* = 43) and Mild (*n* = 74) categories. In contrast, most misclassifications occurred between the Moderate (*n* = 40) and Severe (*n* = 54) classes, reflecting increased visual overlap in advanced bone loss. FocalNet-Tiny correctly classified 41 of 43 Healthy cases and 37 of 40 Moderate cases. CAFormer-S18 and ConvNeXt-Tiny achieved the highest correct counts in the Mild class (67/74 and 65/74, respectively). Misclassification of Severe cases as Moderate was most frequent in CAFormer-S18 (17 cases), FocalNet-Tiny (13 cases), and MaxViT-Tiny (13 cases), whereas ConvNeXt-Tiny, DaViT-Tiny, and EfficientViT-B1 correctly identified the most Severe cases (41/54 each). Validation results and computational costs are summarized in Table 1.

As shown in Table 1, FocalNet-Tiny achieved the highest numerical validation performance, reaching 85.71% accuracy and an 84.88% macro F1-score at epoch 8. CAFormer-S18, DaViT-Tiny, and EfficientViT-B1 each recorded a validation accuracy of 81.90%. Notably, EfficientViT-B1 reached an 80.80% validation macro F1-score with only 7.51 million parameters, making it the most compact model. ConvNeXt-Tiny and MaxViT-Tiny yielded validation macro F1-scores of 77.51% and 77.68%, respectively. Independent test set results are presented in Table 2.

Table 2 shows that FocalNet-Tiny achieved the highest numerical scores on the test set, with 83.41% accuracy, 84.70% balanced accuracy, 84.81% macro precision, and an 83.50% macro F1-score. ConvNeXt-Tiny ranked second (82.94% accuracy and 82.73% macro F1-score), followed by MaxViT-Tiny and DaViT-Tiny (81.04% accuracy each). These findings indicate that modern convolutional, hybrid, and focal modulation backbones all provide strong multi-class discrimination. Advanced evaluation and calibration metrics are presented in Table 3.

**Figure 4:**
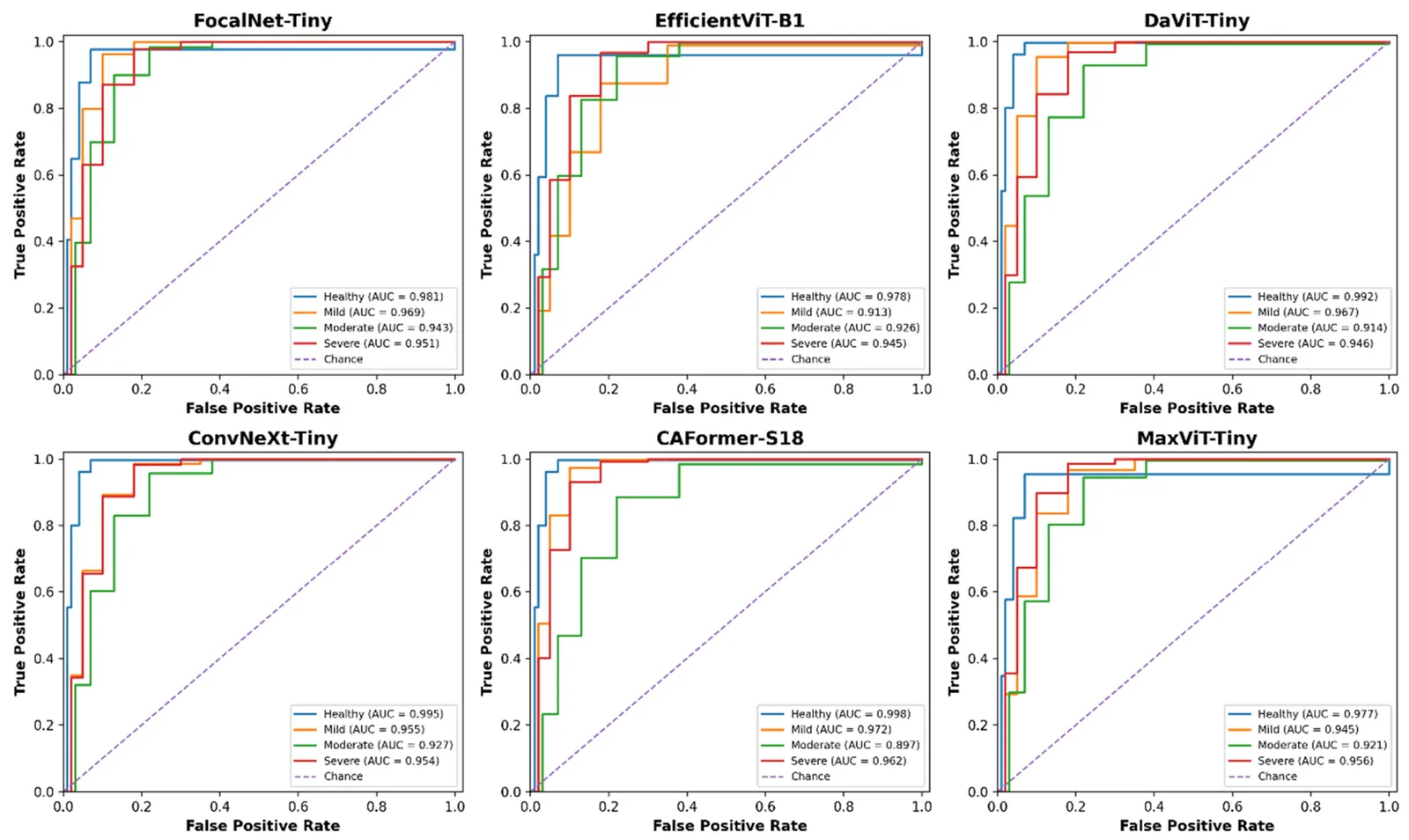
ROC curves of the evaluated models for periodontal bone loss classification.

Table 3 summarizes the agreement, discrimination, and calibration metrics across all six architectures. FocalNet-Tiny recorded the highest numerical values for MCC (0.7842), Cohen’s Kappa (0.7768), QWK (0.8926), and OvR AUC (0.9610). Meanwhile, CAFormer-S18 achieved the best probability calibration with the lowest ECE (0.0438) and lowest Log-Loss (0.4574), as well as the second-highest QWK (0.8861). ConvNeXt-Tiny achieved the second-highest MCC (0.7672) and OvR AUC (0.9578). Overall, all six models demonstrated strong ordinal agreement (QWK: 0.7994–0.8926) and low calibration error (ECE: 0.0438–0.0655). Class-specific ROC curves are presented in Figure 4.

Table 4 presents the results of pairwise McNemar tests conducted to evaluate the performance differences among the six fundamental architectures in greater detail. The *p*-values obtained from the comparisons of the models were all above 0.05, indicating that the classification performances of the evaluated models are quite similar. Notably, FocalNet-Tiny demonstrated superior results in terms of classification accuracy and other performance metrics. These findings support the selection of FocalNet-Tiny as the primary model for this study.

To assess the robustness of the point estimates to sampling variation, bootstrap 95% confidence intervals were computed for all models as described in Section 2.6. The results are presented in Table 5.

The 95% CI for accuracy spanned approximately 10 percentage points, reflecting the moderate size of the test set. These ranges indicate consistent performance. This is also consistent with the McNemar test results presented in Table 4. The class-specific precision, recall, and F1-score values for the FocalNet-Tiny model are detailed in Table 6.

Table 6 shows that FocalNet-Tiny achieved high performance in the Healthy (F1-score: 95.35%) and Mild (F1-score: 85.92%) categories. By contrast, lower scores occurred in the Moderate (precision: 61.67%, recall: 92.50%, F1-score: 74.00%) and Severe (precision: 92.50%, recall: 68.52%, F1-score: 78.72%) classes. Class-wise F1-scores calculated from Figure 3 for the remaining five architectures showed a similar pattern alongside distinct class-level strengths. In the Healthy class, FocalNet-Tiny (95.35%) and ConvNeXt-Tiny (93.98%) achieved the highest F1-scores, followed by DaViT-Tiny and CAFormer-S18 (93.02% each). In the Mild class, CAFormer-S18 led with an F1-score of 86.45%, closely followed by FocalNet-Tiny (85.92%), MaxViT-Tiny (85.91%), and ConvNeXt-Tiny (85.53%). In the Moderate class, ConvNeXt-Tiny (74.07%) and FocalNet-Tiny (74.00%) achieved the top F1-scores, followed by EfficientViT-B1 (72.94%). In the Severe class, EfficientViT-B1 achieved the highest F1-score (79.61%), ahead of FocalNet-Tiny (78.72%), MaxViT-Tiny (77.42%), ConvNeXt-Tiny (77.36%), and DaViT-Tiny (77.36%). Thus, all six architectures reliably separated healthy and mild cases, whereas distinguishing moderate from severe bone loss proved challenging across all model families.

## 4. Discussion

This study benchmarked six modern deep learning backbones—ConvNeXt-Tiny, MaxViT-Tiny, EfficientViT-B1, FocalNet-Tiny, CAFormer-S18, and DaViT-Tiny—under identical training protocols for four-class radiographic periodontal bone loss grading on panoramic ROIs. FocalNet-Tiny achieved the highest numerical accuracy (83.41%), balanced accuracy (84.70%), macro F1-score (83.50%), and OvR AUC (0.9610), while performing in a statistically comparable range to competing architectures such as ConvNeXt-Tiny (82.94%) and CAFormer-S18 (80.57%). Compared with the 76–81% diagnostic concordance reported among dentists in routine radiographic assessments [8], these deep learning models show strong potential for assistive pre-screening, pending multi-center external validation. Key prior studies on artificial intelligence (AI)-based periodontal imaging are compared with the present work in Table 7.

Shon et al. reported 92.9% accuracy in four-stage classification using a multi-stage framework that combined tooth segmentation and keypoint ratio calculation [21]. In our benchmark, single-stage classifiers—led numerically by FocalNet-Tiny (83.41%) and ConvNeXt-Tiny (82.94%)—provided informative severity grading directly from radiographic images without requiring tooth-by-tooth segmentation masks. Avoiding multi-stage annotation pipelines simplifies model development and accelerates pre-diagnostic screening. In periapical radiographs, Lee et al. reported binary classification accuracies of 81.0% for premolars and 76.7% for molars [22]. Using a VGG-16 network on panoramic images, Alotaibi et al. achieved 73.0% accuracy for binary classification and 59.4% for four-class severity grading [13]. The substantially higher four-class accuracies achieved across all six backbones in our study (79.15–83.41%) reflect the improved representation capacity of modern convolutional, transformer, and hybrid architectures. Similarly, the 0.930 AUC reported by Tao et al. for periodontitis screening from intraoral photographs underscores the broader utility of deep learning in periodontal diagnostics [23].

Earlier radiographic studies also align with our results. Krois et al. [8] trained CNNs on manually cropped panoramic segments for binary bone loss detection and achieved 81.0% accuracy, matching experienced examiners. Our study extends this approach from binary detection to four-tier severity grading. Furthermore, Kurt-Bayrakdar et al. [7] reported an AUC of 0.951 for total alveolar bone loss detection using U-Net segmentation. The high OvR AUC values achieved across all six models in our study (0.9481–0.9610) demonstrate that modern classification backbones capture discriminative periodontal context directly from ROI images without pixel-level annotation.

Many recent studies rely on complex multi-stage detection pipelines to stage periodontal bone loss. For example, Jiang et al. [24] combined U-Net and YOLOv4 across 640 panoramic radiographs and reported an overall accuracy of 77.0%, whereas Kong et al. [25] designed a custom two-stage detector. By contrast, single-stage backbones such as FocalNet-Tiny (27.66 M parameters, 83.41% accuracy), ConvNeXt-Tiny (27.82 M parameters, 82.94% accuracy), and the lightweight EfficientViT-B1 (7.51 M parameters, 79.15% accuracy) offer compact alternatives suitable for clinical hardware. The slight numerical edge of FocalNet-Tiny across aggregate metrics suggests that multi-scale focal modulation [26] effectively captures both fine anatomical landmarks (such as the CEJ, lamina dura, and alveolar crest) and broader interdental context [26,27,28,29]. At the same time, the comparable performance of ConvNeXt-Tiny and CAFormer-S18 aligns with recent systematic reviews showing that modernized CNNs and vision transformers achieve similar diagnostic accuracy in medical imaging [30,31].

Beyond classification accuracy, probability calibration is critical because deep neural networks often produce overconfident predictions in medical imaging [32]. All six models in our benchmark exhibited low calibration error, led by CAFormer-S18 (ECE = 0.0438), MaxViT-Tiny (ECE = 0.0524), DaViT-Tiny (ECE = 0.0601), ConvNeXt-Tiny (ECE = 0.0606), EfficientViT-B1 (ECE = 0.0650), and FocalNet-Tiny (ECE = 0.0655). Combining McNemar’s test with bootstrap confidence intervals also provided a rigorous statistical evaluation [20]. Bootstrap resampling confirmed that FocalNet-Tiny produced stable test estimates (accuracy 95% CI: 78.20–88.15%), whereas McNemar’s test showed that its numerical lead over the other five architectures was not statistically significant (*p* > 0.05). Rather than supporting the superiority of a single model, these results demonstrate that multiple contemporary backbone families achieve comparably robust performance under standardized training conditions.

Panoramic radiography remains the standard initial screening modality in periodontology due to its full-mouth coverage, low radiation dose, and cost-effectiveness [33]. Although periapical radiographs and Cone-Beam Computed Tomography (CBCT) offer higher spatial resolution and three-dimensional (3D) detail for assessing angular defects and furcation lesions, panoramic imaging is indispensable for routine baseline evaluation [7,34]. All six architectures in this study achieved OvR AUC values above 0.948, demonstrating resilient feature extraction despite the lower resolution and anatomical overlapping inherent to panoramic images [26].

Class-specific evaluation (Table 6 and Figure 3) revealed that misclassifications across all models occurred primarily between the Moderate (25–50%) and Severe (>50%) categories. In FocalNet-Tiny, for instance, the Moderate class showed 61.67% precision (92.50% recall) and the Severe class showed 68.52% recall (92.50% precision), as 13 Severe cases were predicted as Moderate. Differentiating moderate (mid-root) from severe (apical-half) bone loss on two-dimensional (2D) panoramic radiographs presents several anatomical challenges. First, 2D projection compresses 3D alveolar morphology into a single plane; intact buccal or lingual cortical plates can superimpose over deep intrabony defects and mask the true apical extent of bone loss [7,33,34]. Second, in the posterior maxilla, overlapping of the maxillary sinus floor, zygomatic process, and hard palate obscures both the CEJ and root apices, complicating exact root-length ratio estimation. Third, furcation involvement and root convergence in multi-rooted molars create shadow patterns that mimic moderate horizontal bone loss. Finally, because alveolar bone resorption is a continuous biological process, borderline defects near the 50% threshold (e.g., 45–55% bone loss) share nearly identical radiographic features, leading to natural inter-class overlap for both clinicians and deep learning models.

**Figure 5:**
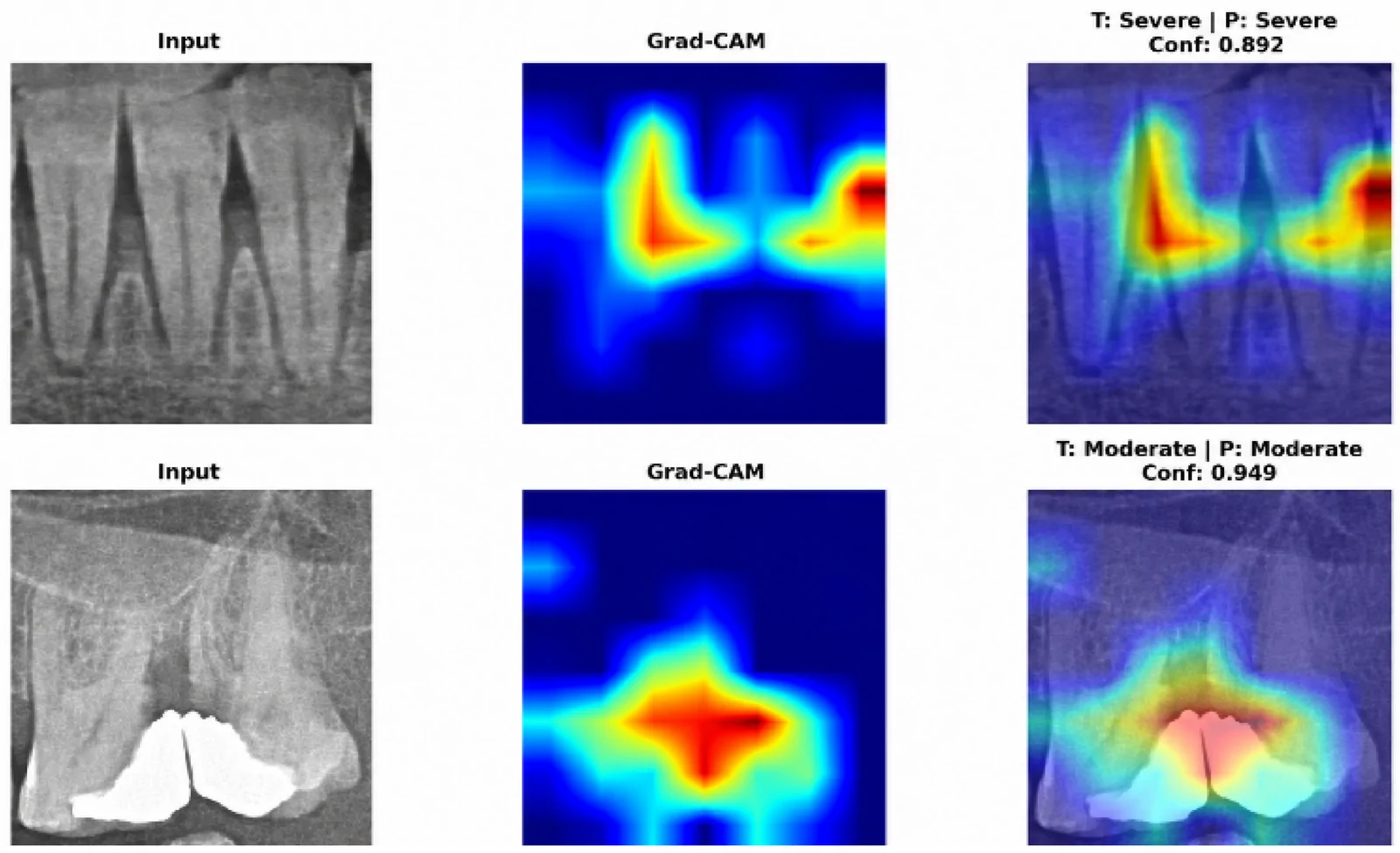
Grad-CAM activation maps of the FocalNet-Tiny model for Severe and Moderate bone loss cases.

Gradient-weighted Class Activation Mapping (Grad-CAM) visualizations were generated to improve model interpretability and clinical trust [35]. The resulting activation maps confirm that FocalNet-Tiny consistently attends to clinically relevant landmarks—specifically the alveolar crest and root surfaces—when grading bone loss severity. Such visual explanations mitigate the black-box nature of deep neural networks and allow clinicians to cross-check automated predictions against anatomical findings (Figure 5).

Several limitations should be considered when interpreting these findings. First, all 1053 images were acquired at a single center using a single panoramic unit (CS 8100 [Carestream Dental LLC, Atlanta, GA, USA]; 68 kV, 8 mA, 10.8 s), and external validation was not performed. Panoramic systems from different manufacturers vary in sensor technology, focal trough geometry, exposure protocols, and contrast profiles. Consequently, the evaluated models cannot yet be deployed as standalone clinical decision support tools without multi-center, multi-vendor external validation. Second, the reference standard was purely radiographic. Because this retrospective dataset lacked chairside clinical records (clinical attachment loss, probing pocket depth, bleeding on probing, and history of tooth loss due to periodontitis), full staging and grading under the 2017 World Workshop framework [18] could not be performed. This has several implications. (i) The four categories reflect radiographic bone loss severity and should not be interpreted as a diagnosis of periodontitis or as equivalent to Stages I–IV. The thresholds used here (<25%, 25–50%, >50%) do not coincide with the 2017 staging cut-offs (Stage I, <15%; Stage II, 15–33%; Stages III–IV, extending to the middle third of the root and beyond), and the distinction between Stages III and IV depends on complexity factors and tooth loss that cannot be assessed radiographically. (ii) Radiographic bone loss reflects cumulative past destruction rather than current disease activity. Without probing and bleeding scores, a reduced but stable periodontium in a successfully treated patient cannot be distinguished from active periodontitis. Similarly, the “Healthy” class indicates the absence of radiographic bone loss and may include patients with gingivitis, which by definition does not involve bone loss. (iii) Alveolar bone loss of non-periodontal origin, such as endodontic lesions, occlusal trauma, orthodontic tooth movement, or iatrogenic causes, may be indistinguishable from periodontal bone loss on panoramic images. (iv) Periodontitis grade (A–C), which requires the bone loss/age ratio and risk factors such as smoking and diabetes, and disease extent (localized vs. generalized), which requires tooth-level assessment, could not be determined because each image was labeled according to its most severely affected tooth. (v) Two-dimensional radiographs tend to underestimate early bone loss relative to clinical attachment loss [33], so incipient lesions may be under-represented in the Mild category. Consequently, the evaluated models should be regarded as tools for radiographic bone loss pre-screening that complement, rather than replace, a comprehensive clinical periodontal examination. Third, because models were trained on manually cropped ROIs rather than full panoramic radiographs, an automated tooth or ROI detection stage would be needed for end-to-end clinical workflows. Fourth, the reference standard relied on specialist visual consensus on 2D radiographs rather than CBCT or intrasurgical measurements. Future studies should prioritize multi-center external validation across diverse X-ray units and integrate clinical parameters with automated full-arch detection.

## 5. Conclusions

This study presented a controlled benchmark of six modern deep learning backbones (ConvNeXt-Tiny, MaxViT-Tiny, EfficientViT-B1, FocalNet-Tiny, CAFormer-S18, and DaViT-Tiny) for four-class radiographic periodontal bone loss grading on panoramic ROIs. Under standardized training conditions, FocalNet-Tiny achieved the highest numerical scores across primary metrics—including accuracy (83.41%), balanced accuracy (84.70%), macro F1-score (83.50%), MCC (0.7842), QWK (0.8926), and OvR AUC (0.9610)—and showed stable estimates in bootstrap analysis. However, McNemar’s test indicated no statistically significant differences in error rates between FocalNet-Tiny and the other five architectures (*p* > 0.05), demonstrating that contemporary convolutional, transformer, and hybrid backbones perform comparably on this task. Across all models, misclassifications occurred mainly between Moderate (25–50%) and Severe (>50%) bone loss due to 2D anatomical superposition. Modern deep learning backbones show promising potential for assistive radiographic pre-screening of periodontal bone loss. However, because all models were developed and tested on single-center, single-device data without clinical periodontal parameters, they are not yet suitable for clinical implementation. Prospective, multi-center external validation across different panoramic systems, together with integration of chairside clinical parameters, is required before these models can be used to support periodontal diagnosis in practice.

## Figures and Tables

**Table 1:** Validation performances and computational costs of the models.

Model	Optimal Epoch	Val. Acc. (%)	Val. Macro-F1 (%)	Training Time (s)	Parameters (M)
FocalNet-Tiny	8	85.71	84.88	202.61	27.66
EfficientViT-B1	15	81.90	80.80	209.34	7.51
CAFormer-S18	10	81.90	81.10	195.33	24.30
DaViT-Tiny	10	81.90	80.77	159.90	27.59
ConvNeXt-Tiny	11	79.05	77.51	189.69	27.82
MaxViT-Tiny	12	79.05	77.68	263.29	30.41

Abbreviations: Optimal Epoch, epoch of peak validation performance (FocalNet-Tiny stopped early at epoch 14 with patience = 6; all other models ran for 15 epochs); Val. Acc. (%), Validation Accuracy; Val. Macro-F1 (%), Validation Macro F1-score; s, seconds; Parameters (M), trainable parameters in millions.

**Table 2:** Test c backbone architectures.

Model	Accuracy (%)	Balanced Accuracy (%)	Macro Precision (%)	Macro Recall (%)	Macro F1-Score (%)	Weighted F1-Score (%)
FocalNet-Tiny	83.41	84.70	84.81	84.70	83.50	83.74
ConvNeXt-Tiny	82.94	82.37	83.21	82.37	82.73	82.99
MaxViT-Tiny	81.04	80.92	81.35	80.92	80.34	81.18
DaViT-Tiny	81.04	80.68	80.40	80.68	80.50	81.18
CAFormer-S18	80.57	79.59	80.70	79.59	79.58	80.54
EfficientViT-B1	79.15	78.98	78.76	78.98	78.76	79.23

**Table 3:** Advanced evaluation metrics of evaluated backbone architectures.

Model	MCC	Cohen’s Kappa	QWK	ECE	AUC (OvR)	AUC (OvO)	Log-Loss
FocalNet-Tiny	0.7842	0.7768	0.8926	0.0655	0.9610	0.9609	0.4814
ConvNeXt-Tiny	0.7672	0.7669	0.8635	0.0606	0.9578	0.9580	0.4696
MaxViT-Tiny	0.7471	0.7431	0.8594	0.0524	0.9501	0.9496	0.5319
DaViT-Tiny	0.7428	0.7425	0.8693	0.0601	0.9546	0.9541	0.4986
CAFormer-S18	0.7383	0.7349	0.8861	0.0438	0.9570	0.9547	0.4574
EfficientViT-B1	0.7171	0.7165	0.7994	0.0650	0.9481	0.9487	0.5386

Abbreviations: MCC, Matthews Correlation Coefficient; Cohen’s Kappa, unweighted kappa coefficient; QWK, Quadratic Weighted Kappa; ECE, Expected Calibration Error; AUC (OvR), One-vs-Rest Area Under the Receiver Operating Characteristic Curve; AUC (OvO), One-vs-One Area Under the Receiver Operating Characteristic Curve; Log-Loss, multi-class cross-entropy logarithmic loss.

**Table 4:** Pairwise McNemar Test Results for Six Models.

Model A	Model B	A Correct/B Wrong	B Correct/A Wrong	McNemar χ^2^	*p*-Value
FocalNet-Tiny	ConvNeXt-Tiny	16	15	0.0000	1.0000
FocalNet-Tiny	MaxViT-Tiny	19	14	0.4848	0.4862
FocalNet-Tiny	EfficientViT-B1	24	15	1.6410	0.2002
FocalNet-Tiny	CAFormer-S18	24	18	0.5952	0.4404
FocalNet-Tiny	DaViT-Tiny	19	14	0.4848	0.4862
ConvNeXt-Tiny	MaxViT-Tiny	18	14	0.2812	0.5959
ConvNeXt-Tiny	EfficientViT-B1	22	14	1.3611	0.2433
ConvNeXt-Tiny	CAFormer-S18	24	19	0.3721	0.5419
ConvNeXt-Tiny	DaViT-Tiny	14	10	0.3750	0.5403
MaxViT-Tiny	EfficientViT-B1	22	18	0.2250	0.6353
MaxViT-Tiny	CAFormer-S18	22	21	0.0000	1.0000
MaxViT-Tiny	DaViT-Tiny	20	20	0.0000	1.0000
EfficientViT-B1	CAFormer-S18	23	26	0.0816	0.7751
EfficientViT-B1	DaViT-Tiny	19	23	0.2143	0.6434
CAFormer-S18	DaViT-Tiny	22	23	0.0000	1.0000

**Table 5:** 95% Confidence Intervals for the Performance Metrics of Six Models.

Model	Accuracy (%)	AUC-OVR	Balanced Accuracy (%)	MCC	Macro-F1 (%)
FocalNet-Tiny	78.20–88.15	0.9410–0.9773	80.08–89.16	0.7192–0.8460	78.37–88.18
ConvNeXt-Tiny	77.73–87.68	0.9355–0.9765	76.94–87.78	0.6962–0.8344	77.26–87.69
MaxViT-Tiny	75.83–86.26	0.9283–0.9690	75.76–85.99	0.6773–0.8167	74.73–85.51
EfficientViT-B1	73.46–84.36	0.9257–0.9663	73.17–84.36	0.6422–0.7891	72.76–84.03
CAFormer-S18	74.88–85.78	0.9382–0.9732	74.01–84.87	0.6629–0.8085	73.84–84.78
DaViT-Tiny	75.83–86.26	0.9344–0.9721	75.00–86.03	0.6690–0.8095	74.75–85.62

**Table 6:** Class-based performance metrics of the FocalNet-Tiny model.

Class	Precision % (Test)	Recall % (Test)	F1-Score % (Test)	Support
Healthy	95.35	95.35	95.35	43
Mild	89.71	82.43	85.92	74
Moderate	61.67	92.50	74.00	40
Severe	92.50	68.52	78.72	54
Macro Average	84.81	84.70	83.50	211
Weighted Average	86.26	83.41	83.74	211

**Table 7:** Comparison of AI-based key studies in periodontal imaging with the current study.

#	Study (Author)	Imaging Method	Classification Task/Number of Stages	AI Architecture	Primary Performance Metrics
1	Kurt-Bayrakdar et al. [7]	Panoramic	Bone Loss Pattern Detection (Segmentation)	U-Net	AUC: 0.951 (Total bone loss)
2	Krois et al. [8]	Panoramic	Binary Classification (Bone loss present/absent)	CNN	Accuracy: 81.0%
3	Alotaibi et al. [13]	Panoramic	Binary and 4-Stage Severity Classification	VGG-16	Accuracy: 73.0% (Binary), 59.4% (Four-class)
4	Shon et al. [21]	Panoramic	4-Stage Periodontitis Classification	YOLOv5 (Segmentation integrated)	Accuracy: 92.9%
5	Lee et al. [22]	Periapical	Binary Classification (Premolar and Molar)	VGG-19	Accuracy: 81.0% (Premolar), 76.7% (Molar)
6	Tao et al. [23]	Intraoral Photograph	Stage II–IV Periodontitis Screening	ResNet50	AUROC: 0.930
7	Jiang et al. [24]	Panoramic	4-Stage Bone Loss Classification	U-Net + YOLOv4	Accuracy: 77.0%
8	Kong et al. [25]	Panoramic	Bone Loss and Severity Detection	Two-Stage Bespoke Detector	Multi-stage bespoke object detection pipeline
9	Current Study	Panoramic	4-Category Radiographic Bone Loss Grading (Healthy, Mild, Moderate, Severe)	FocalNet-Tiny	Accuracy: 83.41%, Macro-F1: 83.50%, AUC: 0.961

#: Indicates the number of studies in the list.

## Data Availability

Data used in this research are available upon reasonable request from the corresponding author.
